# Inline cartridge extraction for rapid brain tumor tissue identification by molecular profiling

**DOI:** 10.1038/s41598-019-55597-7

**Published:** 2019-12-12

**Authors:** Stanislav I. Pekov, Vasily A. Eliferov, Anatoly A. Sorokin, Vsevolod A. Shurkhay, Evgeny S. Zhvansky, Alexander S. Vorobyev, Alexander A. Potapov, Eugene N. Nikolaev, Igor A. Popov

**Affiliations:** 1 Moscow Institute of Physics and Technology, Dolgoprudny, Moscow Region Russian Federation; 2 Federal State Autonomous Institution «N.N. Burdenko National Scientific and Practical Center for Neurosurgery» of the Ministry of Healthcare of the Russian Federation, Moscow, Russian Federation; 3 Skolkovo Institute of Science and Technology, Skolkovo, Russian Federation

**Keywords:** Lipidomics, High-throughput screening, Mass spectrometry, Surgical oncology, Mass spectrometry

## Abstract

The development of perspective diagnostic techniques in medicine requires efficient high-throughput biological sample analysis methods. Here, we present an inline cartridge extraction that facilitates the screening rate of mass spectrometry shotgun lipidomic analysis of tissue samples. We illustrate the method by its application to tumor tissue identification in neurosurgery. In perspective, this high-performance method provides new possibilities for the investigation of cancer pathogenesis and metabolic disorders.

## Introduction

Surgery is one of the essential steps of brain tumor treatment. The surgery outcome is directly connected with the precision of tumor border determination, which requires accurate tissue identification. The volume of tumor resection remains the most important prognostic factor for patient survival and long-term follow-up, as incomplete excision may lead to tumor recurrence, but excessive tissue removal may drastically decrease patient quality of life. At this moment, a variety of navigation systems are used in neurosurgery: preoperative neuroimaging is widespread, but the ‘brain shift’ phenomenon limits its accuracy; intraoperative imaging using MRI or CT is very useful but is time-consuming and not cost-effective. Other techniques such as ultrasound diagnostics or fluorescent labeling also have certain limitations and do not allow for a comprehensive representation of the tissue or are even inapplicable for some tumor types^1–4^. Histopathology is widely used for intraoperative tumor boundary identification, but it is a labor- and, especially, time-consuming process that forces the surgeon to pause the resection process and increase operation time^5^. On the other hand, accurate tumor tissue identification is required for the refinement of postoperative treatment, which significantly influences the disease-free time and the overall survival time. The immunohistochemical methods are currently a gold standard for tumor tissue identification, but in most cases, it takes considerable time for the analysis^6^.

An alternative approach for tumor tissue identification is based on the investigation of the molecular composition of tissues using various metabolites as cancer biomarkers^7–11^. In contrast to specific cancer-related alterations in the genome and proteome, metabolic reprogramming occurring during malignancy^12^ affects the plurality of nonspecific molecular pathways in cells. Prominent changes in energy metabolic pathways of malignant cells lead to intensive aerobic glycolysis^13^ (Warburg effect) required to generate enough ATP^14^ and to the activation of *de novo* lipogenesis^15^, which is proposed as a crucial step in cell proliferation^16^. Alterations in the uptake (which is limited by the hypovascularity of cancer tissue), synthesis (by activation of *de novo* synthesis) and consumption (including beta-oxidation pathways) of various fatty acids in malignant cells^17–19^ result in substantial changes in the cell lipid composition; therefore, many attempts are made to implement lipid profiling for tumor tissue boundary determination^15–17,20–23^. Mass-spectrometric molecular profiling and identification of tissue is now rapidly developed worldwide due to its high sensitivity and versatility^24–28^. Regarding brain tumors and neurosurgery, the most important methods are based on ambient mass spectrometry, or so-called direct tissue analysis, especially on desorption electrospray ionization^11,25^, rapid evaporative ionization^29^ or microextraction followed by electrospray ionization^30–32^. Despite the high precision of these methods, they require time-consuming sample preparation or a high amount of the tissue for analysis, so a simple, rapid and reliable method for direct tissue analysis is still required to transfer mass spectrometry profiling approaches to a clinic.

## Results and Discussion

Inline cartridge extraction (ICE) based on integrating the extraction procedure directly into the solvent flow before the electrospray ionization (ESI) source provides an effective extraction process followed by stable ionization. ICE considerably facilitates sample handling procedures that speed up the entire analysis process. Briefly, the tissue sample is placed inside a cartridge, which then connects directly to the ESI source of the mass spectrometer to minimize the overall dead volume. Separation of the tissue-containing zone and the ESI source not only simplifies the analysis procedure and reduces contamination sites inside the sample injection line and ion source but also allows for the easy application of ICE in any mass spectrometer without the necessity to introduce any changes in the instrument construction. Moreover, only simple sample handling training is required for the operator, as all instrument-operating procedures such as spectra registration and calibration remain routine through the use of standard ESI sources.

**Figure 1:**
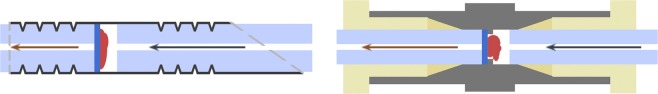
Schematic representation of disposable (left) and reusable (right) cartridges with 1.6 mm ID stainless steel tubing (*black*), stainless steel union (*gray*), 1/16″ OD polymer capillary (*light blue*), glass microfiber filters (*blue*), tissue sample (*red*), and fittings and ferrules (*light yellow*). Arrows represent the direction of the solvent flow.

We developed and tested two types of stainless steel cartridges: disposable and reusable (Fig. 1). Glass microfiber filters (1.2 µm pore size) were applied to protect the ESI source from the sample particles that were washed from the sample surface. Nylon filters should be avoided due to the contamination caused by nylon oligomers. Regenerated cellulose or fluoropolymeric filters are suitable, but glass microfiber filters are much more convenient for cartridge assembly. Both types of preassembled cartridges allow sample loading and fitting to the ion source in approximately one minute. The typical volume of sample loading is approximately 0.5–2 mm^3^, which is sufficient to obtain a stable and intense analytical signal.

The disposable cartridge consists of a 1.6 mm inner diameter (ID) stainless steel tube and a 1/16″ outer diameter (OD) polymer capillary to form the cartridge (see Methods). PTFE (polytetrafluoroethylene) or ETFE (ethylene tetrafluoroethylene) capillaries are preferable, while PFA (perfluoroalkoxy alkane) tubing is not rigid enough to be crimped inside the cartridge body. PEEK (polyether ether ketone) capillaries are also suitable, but polymer contamination might occur in the spectra. These contaminations originate from the end face of the capillaries inside the cartridge, which, in some cases, could not be washed sufficiently even through intensive procedures. Disposable cartridges could be easily assembled even in the operating room if needed, excluding any manipulations with tissues in the laboratory.

The reusable cartridge is based on a stainless steel chromatography union with a 1.25 mm through-hole. Fluoropolymeric 1/16″ OD tubing (PFA, PTFE or ETFE) is required for assembling reusable cartridges to minimize cross-contamination. Reusable cartridges are very cost-effective but require intensive flushing with isopropanol after each analysis and a sonication procedure before assembling (see consumables preparation in Methods chapter).

Each cartridge should be manually purged with the extraction mixture just before the analysis to remove air and traces of blood and normal saline (which is typical for stereotaxic biopsy material). Then, the cartridge is mounted into the electrospray solvent line, and continuous flow of the extraction mixture is applied. We used 90% (v/v) methanol with the addition of 0.1% (v/v) acetic acid as the extraction mixtures, which is optimal for lipid extraction and ionization both in negative and positive modes^27^.

The mismatch identification rate is critically important for the clinical implementation of any diagnostic procedure, and concerning neurosurgery, there is an approximately equal intolerance to false-positive and false-negative identifications. For this reason, not only precise and accurate classifiers are required for the data processing (which requires sufficient statistical data) but prevention of cross-contamination is also crucial. Single-use cartridges significantly reduce cross-sample contamination, but sufficient time for the electrospray source washing procedure after each sample is still required. To improve the performance of the method, we propose an automated procedure that originated from the loop injection^27^ (see Methods). Thus, we reduce the contamination of the injection line and ion source simultaneously with the improvement of washing effectiveness, which increases overall instrument performance. The limiting stage of the analysis is the time required to pass the sufficient volume of the extraction solvent through the cartridge with a relatively slow rate, as a 3- to 5-fold higher solvent flow rate noticeably decreased the component richness of the extract.

**Figure 2:**
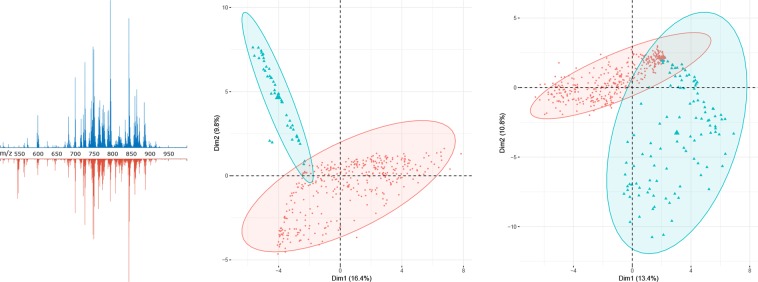
(left) High-resolution mass spectra of glioblastoma (*top*) and astrocytoma (*bottom*) samples, negative mode; (**center**) PCA diagram for astrocytoma (*red*) and nontumor pathological tissue (*blue*), negative mode, low-resolution data; (right) PCA diagram for glioblastoma (*red*) and nontumor pathological tissue (*blue*), positive mode, low-resolution data; a 95% confidence ellipsoid is also shown.

We used our method to profile astrocytoma and glioblastoma samples obtained directly from the operating room. Mass spectrometry profiles of brain tumor tissues (Fig. 2, left) obtained by ICE-MS are suitable for further tissue classification^28^, as ICE closely resembles the extraction followed by ESI and other similar ambient mass spectrometry methods^31,32^ and DESI-MS^25^. However, ICE improves sampling handling procedures and reduces the overall analysis time since no sample preparation or sectioning is required. In addition, ICE is sustainable to ambient conditions such as temperature and humidity in a laboratory and provides the possibility of obtaining a stable ion current in negative mode. Ionization instability expressed as scan-to-scan data variations is an essential issue in ambient mass spectrometry^33–35^, but ICE provides a quite stable signal (mean cosine similarity^33^ (CS) between scans in a sample of 0.977), which is crucial for automated data processing. The ICE-MS molecular profiles of different samples of similar types of tumors are well correlated, but the natural biological variability of tissues is still present in the spectra. To evaluate the reproducibility of the method, we analyzed averaged high-resolution spectra obtained from multiple samples of histopathologically equal tissues resected from a single patient, and for each of five patients, a high CS (0.97–0.99) was observed. The small volume of dissected tissues limited the number of issues included in this investigation; nevertheless, it demonstrates the reproducibility and robustness of the method. For astrocytoma tumor core samples in positive mode, a mean CS of 0.952 was calculated, while for an extended dataset containing astrocytoma and glioblastoma tumor core samples, a mean CS of 0.869 was calculated for low-resolution data, which indicates the possibility to differentiate various tumor types from each other as well as from nontumor pathological tissues.

**Figure 3:**
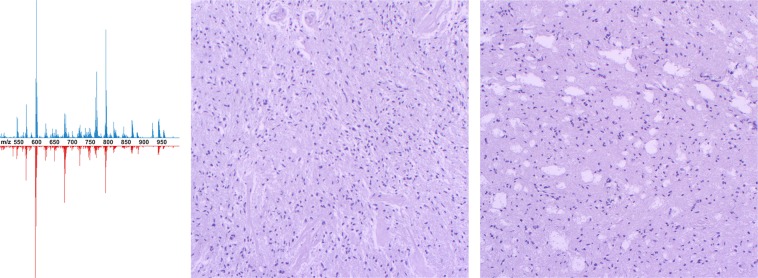
(left) High-resolution mass spectra of the tumor core (*top*) and tumor growth zone (containing 70% malignant cells, *bottom*) of glioblastoma samples in negative mode. Histological images of the tumor core (center) and tumor growth zone (right).

Ambient MS profiles of brain tumor tissues are complex data due to the variety of lipids and metabolites extracted during analysis. However, further statistical analysis and machine learning algorithms allow us to remove the matrix effect and extract features for further tissue differentiation^36^. PCA (Fig. 2, center and right) allow differentiating tumor tissue from the nontumor pathologies used as the control. Low-resolution molecular profiles, both in negative and positive modes, were used for the development of various classifiers with high specificity and sensitivity (Table 1). Classifiers based on high-resolution data demonstrate better specificity, which means a decreased number of false-positive tumor identifications and, in most cases, better overall accuracy. Further including into consideration not only tumor core and control samples but also samples with different percentages of malignant cells (Fig. 3) would allow for the building of regressors required for tumor boundary determination.

**Table 1:** The accuracy (ratio of properly identified samples to all samples), sensitivity (ratio of properly identified tumor samples to all tumor samples) and specificity (ratio of properly identified nontumor pathology samples to all nontumor pathology samples) calculated for classifiers developed separately for each combination of resolution and polarity of data obtained (83% of samples were chosen randomly for a training set by cross-validation).

		Astrocytoma classifiers			Glioblastoma classifiers		
		Accuracy	Sensitivity	Specificity	Accuracy	Sensitivity	Specificity
Low-resolution data	Positive mode	0.993	0.999	0.909	0.985	0.988	0.973
	Negative mode	0.976	1.000	0.916	0.984	0.993	0.949
High-resolution data	Positive mode	0.972	0.982	0.947	0.993	0.995	0.983
	Negative mode	0.986	1.000	0.952	0.988	1.000	0.936

In addition to simultaneous analysis of low- and high-resolution data, which is useful for further instrument-independent implementation of tissue profiling in a clinic, the possibility of analyzing samples after the freeze-thaw cycle was also investigated. Tumor tissue samples obtained from eight different patients were divided into two parts, which were confirmed as histopathologically equal. One half was analyzed in the clinic immediately after resection, and the other half was analyzed in a laboratory after the freeze-thaw cycle. The obtained spectra were similar (median CS of 0.907), and after the unification procedure^37^, high- and low-resolution data could be considered almost identical, which allows for the use of the same classifiers for the analysis of intra- and postoperative samples. The unification procedure provides the possibility to use high-resolution data for low-resolution classifier training and makes ICE suitable for postoperative tissue identification if, for example, high-resolution mass spectrometry profiling is required for diagnosis refinement.

The presented method has broad potential not only for neurosurgery but also for application in various biological and medical fields. As previously shown, ambient mass spectrometry methods provide a reliable method for tissue identification using molecular profile peculiarities in malignant tissue, and ICE is a simple and prospective approach to intraoperative brain tumor tissue identification. From the presented results, we conclude that ICE outperforms previously demonstrated direct tissue analysis methods with the increased performance and essential simplification of sample preparation steps. The possibility of obtaining the results of tissue investigation in a few minutes is especially crucial in the case of multiple stereotactic biopsy samples of highly heterogeneous tumors. From a broad perspective, ICE could be applied as the substitution of different types of express histology in a clinic. Second, the application of mass spectrometry profiling provides a way for further improvement of the accuracy of postoperative tissue analysis required for treatment correction. Detailed tissue investigation is possible not only onsite but also in a distant laboratory using frozen tissue samples. Additionally, ICE high throughput allows for labor-effective profiling of a significant amount of tissue samples that could be used for investigations in the study of carcinogenesis, metabolomic disorders and in clinical studies that require large datasets for statistical analysis. More broadly, the presented work suggests a way to apply rapid molecular profiling in medical diagnostics as well as in further biological research: a simple, universal, time- and cost-effective method for direct tissue analysis.

## Methods

### Reagents

HPLC-grade solvents and reagents were obtained from Merck (Merck KGaA, Darmstadt, Germany).

### Samples

The samples were provided by the N.N. Burdenko NSPCN and analyzed under an approved N.N. Burdenko National Scientific and Practical Center for Neurosurgery (NSPCN) Institutional Review Board protocol. A signed informed consent form, filled out in accordance with the requirements of the local ethical committee and specifically noting that all removed tissues can be used for further research, was obtained from all patients before surgery. The study was conducted in accordance with the Helsinki declaration as revised in 2013. All protocols and procedures were carried out according to the relevant guidelines and regulations approved by N.N. Burdenko NSPCN Institutional Review Board. Fragments of tissues resected during surgery were divided into equal parts, placed in normal saline, and half of each sample was subjected to routine hematoxylin and eosin staining and further immunohistochemical analysis, according to which the samples were classified as glioblastoma (n = 27) or astrocytoma (n = 20) tumor core tissue or nontumor pathological tissue (n = 9). Samples for mass spectrometric analysis were analyzed onsite without delay, or they were frozen and stored at −80 °C until analysis.

### Consumable preparation

The stainless steel capillary (1.6 mm OD) was cut into 3 cm long sections (one end was made diagonal for simplification of sample handling). Capillaries and other stainless steel consumables were washed in acetone and sonicated for 15 minutes in isopropanol followed by drying under nitrogen flow. PFA (1/16″ OD × 0.01″ ID; IDEX Health & Science LLC., Oak Harbor, WA, USA), PTFE (1/16″ OD × 0.25 mm ID; Akvilon, Moscow, Russia) or PEEK (1/16″ OD × 0.005″ ID; IDEX Health & Science LLC) tubing was cut onto 3–5 cm long sections and sonicated in isopropanol for 10 minutes followed by sonication in methanol for 10 minutes. A sheet of Whatman^®^ grade GF/C glass microfiber filter (GE Healthcare, Chicago, IL, USA) was soaked in methanol for 30 minutes and dried under a gentle nitrogen stream. Stainless steel unions (10–32 coned thread port configuration, 1.25 mm through-hole) (IDEX Health & Science LLC.), two-piece finger tight fittings and ferrules (for 10–32 coned thread port configuration and 1/16″ OD capillaries; IDEX Health & Science LLC.) were washed with isopropanol, sonicated sequentially in isopropanol and methanol for 10 minutes each time and were dried under nitrogen flow.

### Disposable cartridge assembly

**Figure 4:**
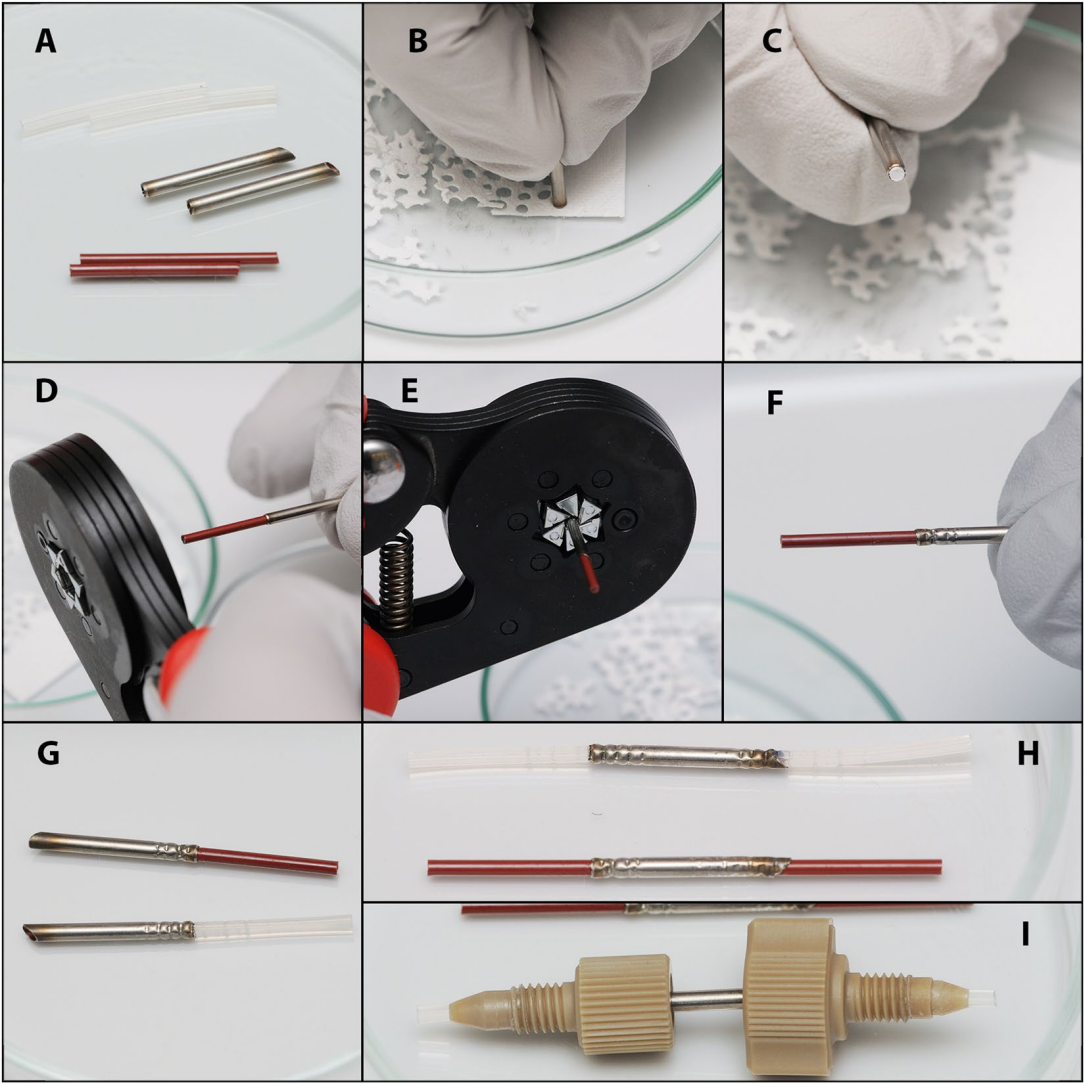
Disposable cartridge assembly. (**A**) prepared consumables; (**D**–**F**) cartridge preassembly; (**G**) preassembled cartridge; (**H**) assembled cartridge with the sample inside; (**I**) cartridge with fittings.

A filter was cut from the glass microfiber filter sheet using the straight end of the stainless steel capillary (Fig. 4, B and C). One polymer (PEEK or PTFE) tubing was inserted into the steel capillary to 2 cm from the sloped end. The filter was pushed inside the cartridge using a second piece of polymer tubing until it touched the first piece of tubing. Then, the second piece of tubing was carefully compressed using the crimp tool (Knipex KN 975314; Knipex-Werk, Wuppertal, Germany), and the first piece of tubing was removed (Fig. 4D–F). The tissue sample was placed into the cartridge from the sloped end using a needle or microspatula and pushed inside to a 15–18 mm depth. Then, the polymer tubing was inserted from the sloped end of the cartridge and compressed as described above (Fig. 4H).

### Reusable cartridge assembly

Filters were cut from the glass microfiber sheet using the straight end of the 1.6″ ID stainless steel capillaries. The capillary with the filter piece was placed inside the coned thread port of the stainless steel union (1.25 mm through-hole), and the filter was pushed into the union using stainless steel wire (1.5 mm OD). After careful capillary removal, the two-piece finger tight fitting with fluoropolymeric (PFA or PTFE)) tubing was connected to the union to press down the filter. The tissue sample was placed into the 1.25 mm ID stainless steel capillary, which was applied to the unoccupied end of the union. The tissue was transferred into the cartridge by pushing it out of the capillary using 1.2 mm OD stainless steel wire. Then, a second filter was applied over the tissue and pressed down with the second ferrule and the fluoropolymeric capillary.

### Extraction and analysis procedure

The cartridge was manually purged with 50 μl of the extraction solvent (90% methanol, 0.1% acetic acid, v/v), visually controlled by the clarity of the eluent. The cartridge with the sample was attached to the inlet of the ESI source, and the solvent flow (3 μl/min) was applied as well as a high voltage. Spectra acquisition was performed with the same instrument parameters that were obtained by routine calibration and tuning procedures in negative and positive modes. Experiments were performed using an LTQ XL ETD (ThermoFisher Scientific, San Jose, CA, USA) or LTQ XL Orbitrap ETD (ThermoFisher Scientific, San Jose, CA, USA) instrument in the clinic or the remote laboratory, respectively. The linear ion trap was operated at the “normal” scan rate, and the FTMS mass resolution was set to 30,000 (FWHM at m/z 400). The spectra were acquired in the ranges of m/z 500–1000 and m/z 100–2000 consequentially.

### Automated analysis procedure setup

The following components were connected to a 6-port divert valve: (port 1) the pump, filled with the extraction solvent (as above) followed by the cartridge; (port 2) outlet to the ESI; (port 3) the pump, filled with the washing solvent (9 μl/min, 65% methanol, 30% isopropanol; 0.1% acetic acid, v/v); ports 4 and 5 were connected via capillary; and (port 6) the outlet to waste. The cartridge was manually purged with 50 μl of the extraction solvent visually controlled by the clarity of the eluent. At the start of the analysis, the divert valve was set at position 1–6, so the first 2 μl of the solvent passed through the cartridge was discarded to waste, and then, the divert valve switched to position 1–2 to deliver the extract into the ESI source. After the extract reached the ion source, the acquisition was initiated, and data were collected for 30 seconds each for negative mode using the LTQ and Orbitrap mass analyzers, followed by a similar positive mode acquisition.

### Post-acquisition procedure

After each acquisition, the union at the ion source inlet (for direct cartridge connection) or port 1 of the divert valve (for automated analysis setup) was flushed with isopropanol. The ion source was washed with the same solvent mixture or washing mixture as above until the intensity of the signal at m/z 600–900 Da decreased by at least 2 orders of magnitude from the level at the end of acquisition.

### Data analysis

Each mass spectrum was averaged over a 5-second window and interpreted as an N-dimensional vector for PCA differentiation and cosine similarity calculation (the comparison of molecular profiles was made by the analysis of mass range m/z 600–1000). Lasso and LDA classifiers were trained using in-home developed software packages with K-fold cross-validation utilizing K = 6 with 20 repetitions.

## Data Availability

The data and software developed for this study are available from the corresponding author upon reasonable request.
